# The N-terminus of RPA large subunit and its spatial position are important for the 5′->3′ resection of DNA double-strand breaks

**DOI:** 10.1093/nar/gkv764

**Published:** 2015-10-10

**Authors:** Margaret Tammaro, Shuren Liao, Jill McCane, Hong Yan

**Affiliations:** Fox Chase Cancer Center, 333 Cottman Avenue, Philadelphia, PA 19111, USA

## Abstract

The first step of homology-dependent repair of DNA double-strand breaks (DSBs) is the resection of the 5′ strand to generate 3′ ss-DNA. Of the two major nucleases responsible for resection, EXO1 has intrinsic 5′->3′ directionality, but DNA2 does not. DNA2 acts with RecQ helicases such as the Werner syndrome protein (WRN) and the heterotrimeric eukaryotic ss-DNA binding protein RPA. We have found that the N-terminus of the RPA large subunit (RPA1N) interacts with both WRN and DNA2 and is essential for stimulating WRN's 3′->5′ helicase activity and DNA2's 5′->3′ ss-DNA exonuclease activity. A mutant RPA complex that lacks RPA1N is unable to support resection in *Xenopus* egg extracts and human cells. Furthermore, relocating RPA1N to the middle subunit but not to the small subunit causes severe defects in stimulating DNA2 and WRN and in supporting resection. Together, these findings suggest that RPA1N and its spatial position are critical for restricting the directionality of the WRN-DNA2 resection pathway.

## INTRODUCTION

DNA double-strand breaks (DSBs) are among the most deleterious damages to the genome. They are repaired by two major types of mechanisms: non-homologous end joining (NHEJ) and homology-dependent repair (HDR) (1–3). The key event in the choice between NHEJ and HDR is the initial processing of DNA ends (4). NHEJ involves limited processing, but HDR requires extensive processing to form long 3′ ss-tails. Recent studies have elucidated two major pathways for the degradation of the 5′ strands of DNA to form 3′ ss-tails. One pathway is catalyzed by the combined actions of a RecQ-type DNA helicase, such as the Werner Syndrome protein (WRN) and the BLM Syndrome protein (BLM), and the DNA2 nuclease (5–9). Detailed mechanistic analyses in *Xenopus* egg extracts showed that WRN uses its 3′->5′ helicase activity to unwind DNA ends and DNA2 then degrades the 5′ ss-tail, leaving the 3′ ss-tail as the product (5,7). The other pathway is catalyzed by a 5′->3′ ds-DNA exonuclease EXO1 (8–10). These two pathways are initiated by the RMX/MRN (Mre11-Rad50-Xrs2/Nbs1) complex and Sae2/CtIP (8,9,11–14).

The most unique feature that sets DSB resection apart from general DNA degradation is the directionality. Resection proceeds in the 5′->3′ direction to degrade only the 5′ strand. EXO1 has an intrinsic 5′->3′ ds-DNA exonuclease, which explains the directionality of this pathway (10). Purified DNA2, however, has no preference for directionality, yet in *Xenopus* egg extracts it degrades only the 5′ ss-tail (5,7,15). The key factor that restricts the directionality of DNA2 and resection is replication protein A (RPA) (7,16–18). RPA is the major eukaryotic ss-DNA binding protein that participates in all transactions involving ss-DNA formation (19,20). RPA consists of three subunits: RPA1, RPA2 and RPA3, that are organized into a highly modular structure. In the presence of RPA only the 5′ strand ss-DNA is degraded (7). Indeed, RPA is essential for the WRN-DNA2 resection pathway and the three proteins together constitute the simplest resection machine that can degrade long DNA molecules at least 5.7 kb in length (18). Yet how RPA restricts the directionality of DNA2 nuclease activity and promotes 5′ strand resection are unclear. A unique feature of RPA binding to ss-DNA is that it is directional, with the N-terminus of RPA1 oriented close to the 5′ end and RPA2 close to the 3′ end (21,22). An appealing hypothesis is that this directional binding is critical for RPA to recruit WRN and DNA2 to their respective substrates to unwind DNA in the 3′->5′ direction and degrade ss-DNA from the 5′ end. Similar hypotheses have been proposed for RPA-interacting proteins involved in nucleotide excision repair and checkpoint response, but have never been tested (21,23,24).

In this study, we addressed this question by dissecting the domain that is critical for RPA to restrict the directionality of DNA2 and promote 5′->3′ resection. We have found that the N terminus of the large subunit of RPA (RPA1N) interacts with both WRN and DNA2. This domain is essential for the stimulation of WRN's helicase activity and the conversion of DNA2 into a 5′->3′ ss-DNA exonuclease. A mutant RPA complex that lacks this N terminal domain is unable to support resection in *Xenopus* egg extracts. In vivo, cells expressing RPA1 that lacks the N terminus are defective in DSB resection. Furthermore, we have found that moving RPA1N to the N terminus of RPA2, but not to the C-terminus of RPA3, causes severe defects in stimulating WRN and DNA2 and in supporting resection. Together, these data suggest that RPA1N as well as its spatial position are critical for the stimulation of WRN and DNA2 to resect DNA in the 5′->3′ direction.

## MATERIALS AND METHODS

### Preparation of *Xenopus* egg extracts and immunodepletion of RPA

Membrane-free cytosolic extracts of unfertilized *Xenopus* eggs were prepared following the published procedure (25). Immunodepletion of RPA from extracts was carried out as previously described (18).

### Cell culture and stable cell line construction

The human osteosarcoma (U2OS) cells were grown in the Dulbecco's modified Eagle's medium (DMEM) supplemented with 10% fetal bovine serum, 2 mM l-glutamine, non-essential amino acids, and penicillin/streptomycin at 37°C in a humidified incubator containing 5% CO_2_. Cells and cell culture reagents were purchased from the Tissue Culture Facility of Fox Chase Cancer Center. Stable cells expressing RPA1 and RPA1N deletion were constructed as follows. The RPA1 open reading frame (ORF) and the RPA1 mutant that misses the first 121 amino acids were subcloned downstream of the CMV promoter in a vector (pDs-NA) that carries the kanamycin resistance gene. The plasmids were digested with the restriction enzyme MluI, which has a unique site outside the kanamycin resistance gene and the RPA1 gene, and then transfected into U2OS cells with Lipofectamine 2000 (Invitrogen, CA). Stable cells were selected with the antibiotic G418 at 1mg/ml concentration.

### Knockdown by siRNAs and indirect immunofluorescence staining

Cells were seeded in 24-well plates containing coverslips at a density of 6000 cells per well. After 24 h, they were transfected with 20nM of control siRNA (D-0012101–03; Dharmacon, CA, USA) or RPA1 siRNA (SI02663696; Qiagen, CA, USA) using HiPerFect (Qiagen, CA, USA) following the manufacturer's protocol. This step was repeated 24 h later. After another 48 h of incubation, cells were treated with 250 μM etoposide for 2 h (EdU was added 15 min prior to etoposide). Cells were fixed and stained for RPA, CenpF and EdU as previously described (26). Images were captured with a monochrome DAGE-MTI cooled CCD-300-RT camera (Scion Corp, MD, USA) and processed for brightness, contrast, and pseudo-colors in Photoshop 5.5 (Adobe Systems, CA, USA).

### Expression and purification of recombinant GST fusion proteins with various regions of *Xenopus* RPA

The genes for the three *Xenopus* RPA subunits and various fragments of RPA1 were subcloned into a pGEX vector to create in-frame fusion proteins with glutathione-S-transferase (GST). Plasmids were introduced into the bacterial strain BL21(DE3) by transformation and fusion proteins were expressed as previously described (27). The fusion proteins were purified with glutathione Sepharose columns (Sigma, MO, USA) following the standard procedure (28). The purified fusion proteins were dialyzed against ELB buffer (10 mM HEPES (pH 7.5)/50 mM KCl/2.5 mM MgCl_2_/250 mM sucrose/1 mM DTT) at 4°C and then stored at –80°C in 5 μl aliquots.

### Expression and purification of recombinant *Xenopus* RPA complexes

The three genes encoding the *Xenopus* RPA subunits were subcloned into two prokaryotic expression vectors: pET-JM for RPA1 and pACYC-Duet (EMD Millipore, MA, USA) for RPA2 and RPA3. For wild-type and 1NΔ RPA, RPA3 contains a His6 tag at the C-terminus. For 3-1N RPA, the His6 tag is at the C-terminus of the fusion subunit. For 1N-2 RPA, the His6 tag is at the N-terminus of RPA1N-RPA2 fusion subunit. To express the three subunit complex, 1 l of BL21(DE3) cells containing the two expression plasmids were grown at 37°C with shaking to OD_600_ 0.4 and then induced with 1 mM IPTG for 3 h. Cells were pelleted and then resuspended in 10 ml of buffer A50 (buffer A (40 mM Tris·HCl (pH 7.5) /1 mM EDTA/1mM DTT/10% glycerol) + 50 mM NaCl) containing 20 mg of lysozyme. After incubation on ice for 1 h, the lysed cells were sonicated and then centrifuged at 10 000 rpm at 4°C for 1 h. The supernatant was first fractionated on a 5 ml HiTrap Ni column (GE Healthcare Life Science, PA, USA) with a gradient from 5 mM imidazole/Tris·HCl (pH 7.5)/0.5 M NaCl to 500 mM imidazole/Tris·HCl (pH 7.5)/0.5 M NaCl. Peak fractions were further fractionated on a 1 ml HiTrap Q column (GE Healthcare Life Science, PA, USA) with a gradient from A50 + 0.005% NP-40 (Roche, NJ, USA) to A500 + 0.005% NP-40. Peak fractions were adjusted to 150 mM NaCl, concentrated, and saved as small aliquots at –80°C.

### Expression and purification of recombinant *Xenopus* DNA2

The expression of *Xenopus* DNA2 with a C-terminal FLAG epitope in SF9 cells and partial purification by HiTrapQ and HiTrap Heparin columns (Sigma, MO, USA) were carried out as previously described (18). 400 μl of DNA2 peak fractions from the Heparin column (at 425 mM NaCl) were incubated with 40 μl of anti-FLAG mAb magnetic beads (Invitrogen, CA, USA) at 4°C for 90 min. After three rounds of washing with 400 μl buffer A supplemented with 150 mM NaCl and 0.005% NP-40, FLAG-DNA2 was eluted by incubating with 0.25 mg/ml FLAG peptide in the same buffer at 4ºC for 30 min. The eluted protein was saved as 2 μl aliquots at –80°C.

### Protein interaction assays

Five microliters glutathione Sepharose beads coated with GST-RPA1N or GST were incubated with 20 μl His-tagged *Xenopus* WRN 1–465 (27), *Xenopus* egg extract (diluted 1:1 with ELB), or FLAG-tagged *Xenopus* DNA2 (in buffer A supplemented with 100mM NaCl and 0.005% NP40) at 4°C for 1 h. The beads were washed twice with 50 μl buffer A supplemented with 150 mM NaCl and 0.005% NP-40. Proteins in the beads and supernatants fractions were analyzed by western blot with antibodies against *Xenopus* WRN and DNA2.

### DNA binding assays

DNA binding assays were carried out in 10 μl reactions containing 15 ng/μl M13mp18 ss-DNA (NEB, MA) and various proteins in ELB. After 30 min of incubation at 4°C, the reactions were analyzed by 1.0% agarose/TAE gel electrophoresis. DNA was detected by staining with SYBR Gold (Invitrogen, CA, USA).

### DNA unwinding, nuclease and resection assays

These assays were performed as previously described (5,7,10,18).

## RESULTS

### The N terminus of RPA1 interacts with both WRN and DNA2

**Figure 1.**
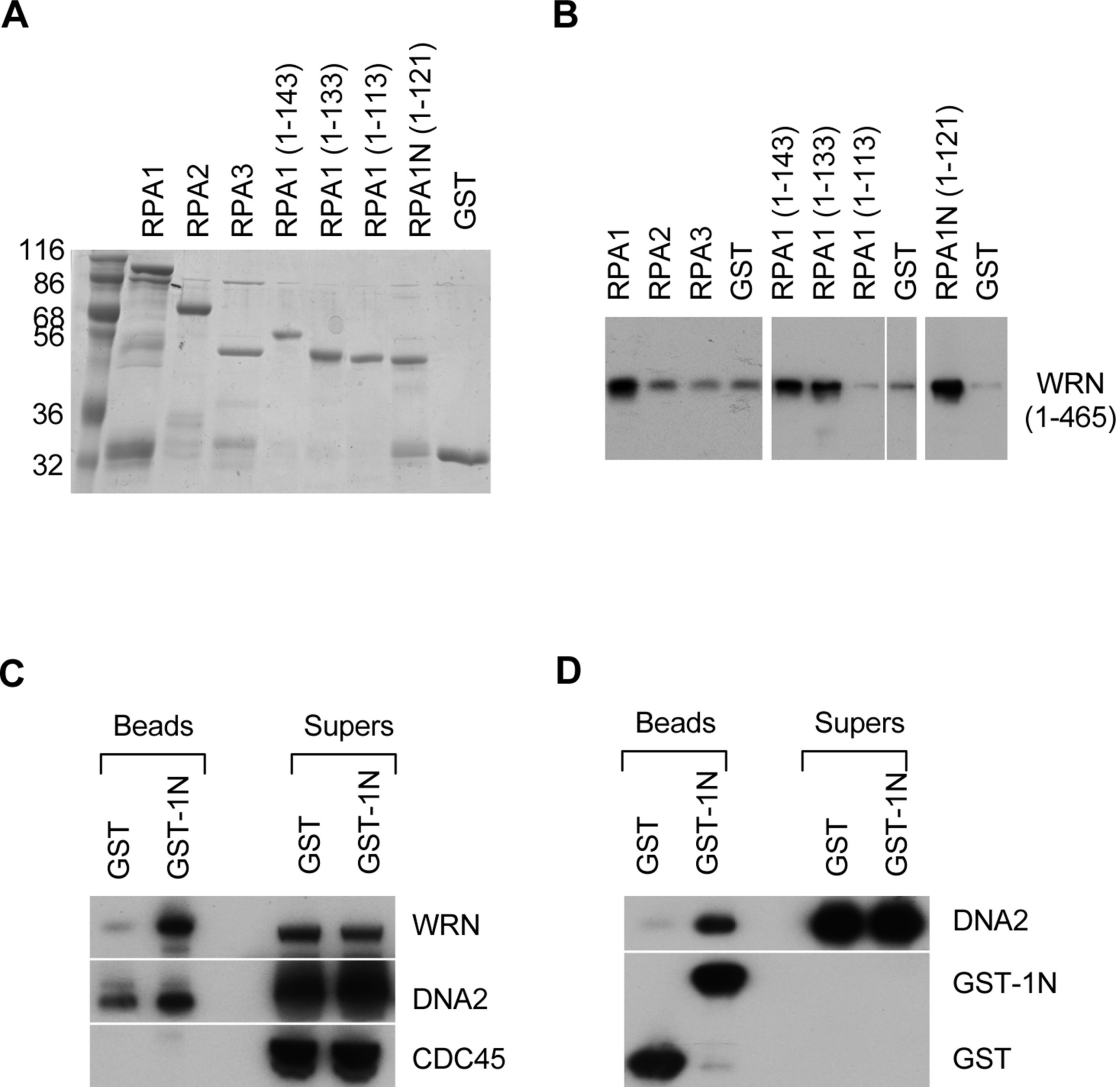
The N-terminal domain of RPA1 interacts with WRN and DNA2. (**A**) A gel showing the GST fusions of various parts of *Xenopus* RPA. The proteins were separated on a 12% SDS-PAGE gel and stained by Coomassie brilliant blue. The size markers are in kilodaltons. (**B**) A Western blot showing the interaction between *Xenopus* WRN and RPA1N. The various GST fusion proteins were incubated with the N-terminal 455 amino acids of WRN and then isolated by glutathione Sepharose beads. The proteins bound to beads were analyzed for WRN by Western blot. (**C**) A western blot showing the interaction between RPA1N (GST-1N) with WRN and DNA2 in *Xenopus* egg extracts. (**D**). A western blot showing the interaction between RPA1N (GST-1N) and DNA2.

The *Xenopus* RPA is known to physically interact with WRN (27). While the N terminal 465 amino acids of WRN have been shown to mediate the interaction, the reciprocal interaction domain on RPA has not been mapped. RPA is a heterotrimeric protein with multiple distinct domains for DNA binding and protein interaction. We first determined which subunit is responsible for *Xenopus* WRN binding. Each subunit of *Xenopus* RPA was expressed as a glutathione S-transferase (GST) fusion protein in *E. coli* and purified (Figure 1A). The fusion proteins were first incubated with WRN1–465 and then pulled down with glutathione Sepharose beads. As shown in Figure 1B, the large subunit, RPA1, could efficiently pull down WRN, suggesting that it mediates the interaction with WRN. The interaction domain was further narrowed down with GST fusion containing different regions of RPA1. The results showed that the N terminal 121 amino acids of RPA1 (RPA1N) is the minimal domain mediating the interaction with WRN. This is consistent with many previous studies showing that this region forms a distinct domain involved not in ss-DNA binding but in interactions with many proteins involved in replication, repair, and checkpoint activation (19,20).

RPA is also known to interact with DNA2 and stimulates its 5′->3′ ss-DNA exonuclease activity (7,18), so we asked if RPA1N mediates the interaction between the two proteins as well. GST-RPA1N was first incubated with *Xenopus* egg extracts and then pulled down with glutathione Sepharose beads. As shown in Figure 1C, RPA1N was indeed able to interact with not only WRN but also DNA2 in extracts. A similar pull-down experiment was carried out with the purified recombinant DNA2 protein. As shown in Figure 1D, RPA1N was also able to interact with the purified DNA2, indicating that the interaction was direct. Together these data showed that RPA1N can interact with both WRN and DNA2.

### RPA1N is important for stimulating the helicase activity of WRN

**Figure 2.**
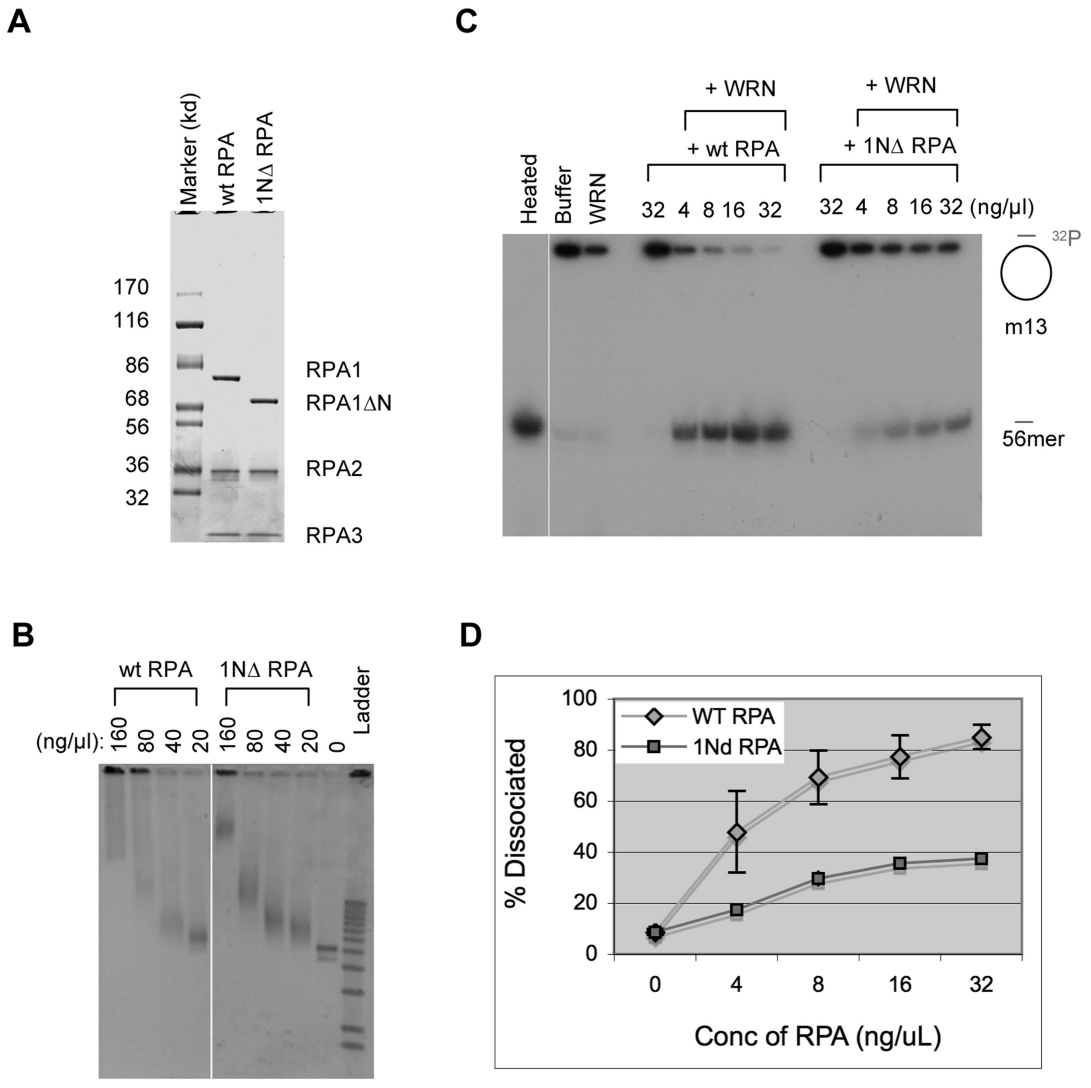
RPA1N is required for the stimulation of WRN's helicase activity. (**A**) A gel showing recombinant *Xenopus* wild-type RPA and mutant RPA lacking RPA1N (1NΔ RPA). The proteins were separated by a 4–12% NUPAGE MOPS gel (Invitrogen, CA) and stained by Coomassie brilliant blue. (**B**) An agarose gel showing the binding activity of the wild-type and 1NΔ RPA to ss-m13 DNA. The binding reactions were separated by a 1% TAE-agarose gel and DNA was detected by staining with SYBR Gold. (**C**) Effect of wild-type RPA and 1NΔ RPA on the unwinding activity of WRN. The substrate was a ^32^P-labeled 56mer oligonucleotide annealed to m13 ss-DNA. After incubation at room temperature for 30 min, the reactions were separated by a 8% TAE-PAGE and DNA was detected by exposure of the dried gel to X-ray film. (**D**) Quantitaton of the unwinding activity in the presence of wild-type RPA or 1NΔ RPA. The percentages of the oligonucleotide dissociated were quantitated and the averages and standard deviations were calculated from three sets of data and plotted.

RPA is known to not only interact with WRN but also stimulate its helicase activity (27). Is RPA1N important for this stimulation? To address this question, we expressed and purified recombinant *Xenopus* RPA that lacked RPA1N (Figure 2A). As expected, this mutant RPA complex (1NΔ RPA) still retained ss-DNA binding activity (Figure 2B). To determine the effect on WRN's helicase activity, various amounts of wild-type or 1NΔ RPA were incubated with WRN, ATP, and a radioactively labeled DNA fragment annealed to m13 ss-DNA. After 30 min of incubation, the reactions were terminated and analyzed by TAE-PAGE. Due to the large size of m13 ss-DNA, only the dissociated labeled fragment could enter the gel. As shown in Figure 2C&D, wild-type RPA could efficiently stimulate the unwinding activity of WRN. In contrast, 1NΔ RPA had only a very small effect, presumably purely due to its binding to ss-DNA to prevent re-annealing of the dissociated DNA. These data showed that the N terminus of RPA1 is indeed important for stimulating the helicase activity of WRN.

### RPA1N is also important for stimulating the 5′->3′ ss-DNA exonuclease activity of DNA2

**Figure 3.**
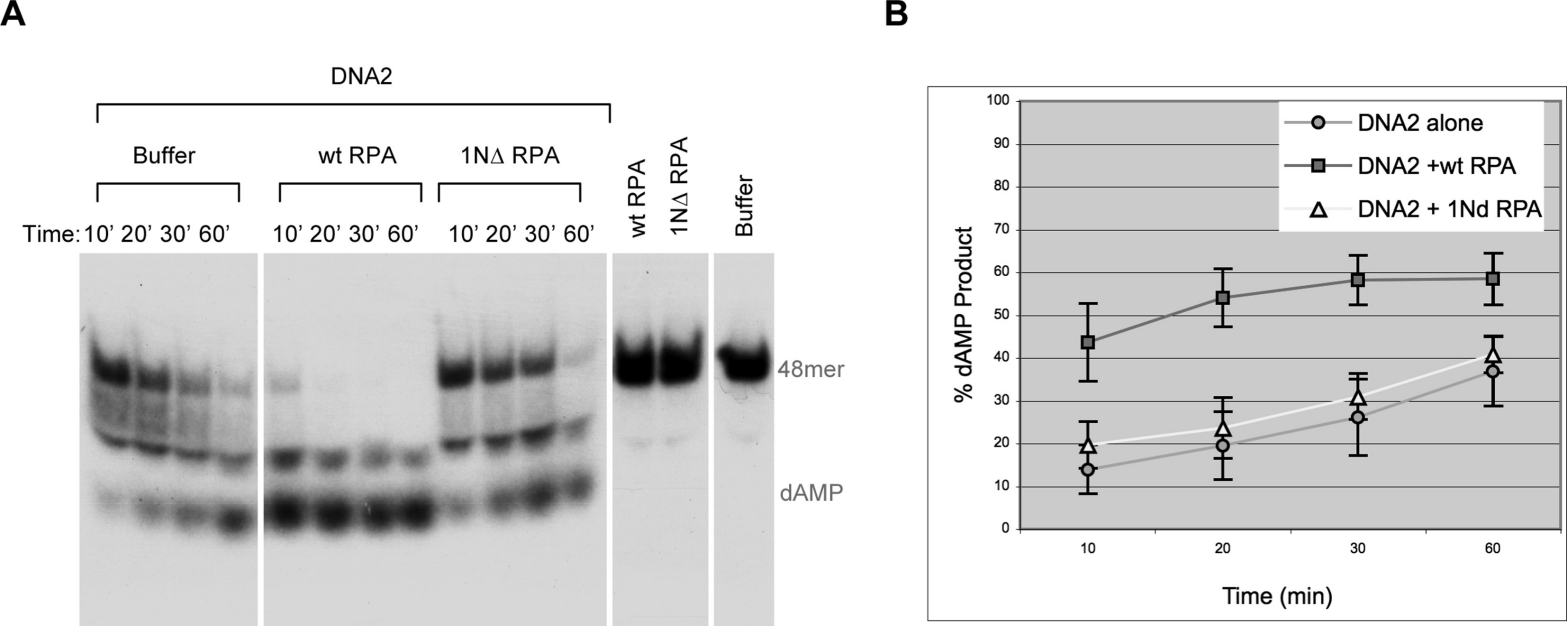
RPA1N is required for the stimulation of DNA2's 5′->3′ exonuclease activity. (**A**) Nuclease assay with a ^32^P-labeled ss-48mer oligonucleotide attached to magnetic beads via a biotinylated nucleotide at the 3′ end of the labeled strand. The substrate was incubated with DNA2 and wild-type or 1NΔ RPA (4 ng/μl) for the indicated times and the products were analyzed by a 8% TAE-PAGE. (**B**) The percentages of nucleotide products were quantitated and the averages and standard deviations were calculated from three sets of data and plotted.

Is RPA1N important for stimulating DNA2's nuclease activity? To address this question, wild-type or 1NΔ RPA were incubated with DNA2 and a ^32^P-labled 48mer ss-oligo attached to Streptavidin magnetic beads via a 3′ biotinylated nucleotide. Products taken at various times were analyzed by TAE-PAGE. As expected, wild-type RPA strongly stimulated the nuclease activity of DNA2 (Figure 3A&B). In contrast, 1NΔ RPA displayed no significant stimulatory activity. Both RPA complexes were active at inhibiting the 3′->5′ degradation of ss-DNA by DNA2, confirming that 1NΔ RPA's lack of stimulation of DNA2's 5′->3′ nuclease activity was not due to defective binding to the ss-oligonucleotide substrate (Supplementary Figure S1A). These results showed that the RPA1N domain is also important for stimulating the 5′->3′ ss-DNA exonuclease activity of DNA2.

### RPA1N is required for resection in *Xenopus* egg extracts

**Figure 4.**
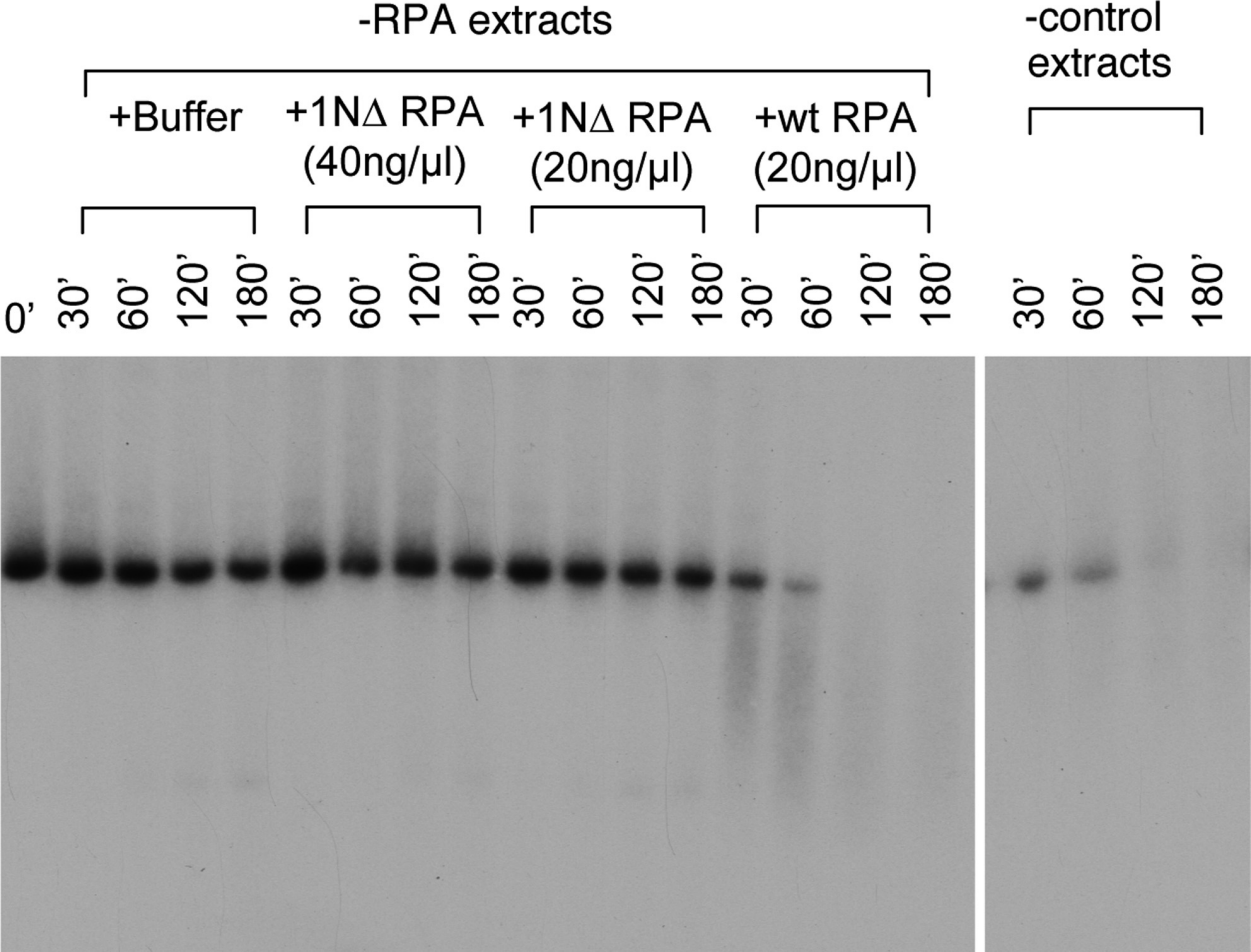
RPA1N is required for resection in *Xenopus* egg extracts. The substrate for resection was a 5.7 kb linearized DNA with a ddC at the 3′ end and a ^32^P label immediately inside. The substrate was incubated with mock depleted or RPA depleted extracts supplemented with various RPA proteins or buffer. Samples taken at the indicated times were analyzed by a 1% TAE-agarose gel.

The enzymatic data strongly argue that the RPA1N domain should be important for resection. To test this hypothesis, we determined if 1NΔ RPA could support resection in *Xenopus* egg extracts. The substrate for the resection assay was a 5.7 kb linear DNA that had a ddCTP at the 3′ end and a ^32^P-dATP immediately inside. We have previously shown that this type of DNA was difficult to repair by NHEJ due to the ddC at the 3′ end and consequently channeled to resection (5). The DNA was incubated in mock or RPA depleted extracts and samples taken at various times were analyzed by TAE agarose gel electrophoresis. As shown previously, DNA was rapidly degraded in mock depleted extracts but very stable in RPA depleted extracts (Figure 4). Addition of the wild-type RPA could efficiently rescue the resection defect of RPA-depleted extracts. In contrast, addition of 1NΔ RPA did not show any significant rescue of resection. These data demonstrated that the RPA1N domain is indeed important for resection in *Xenopus* egg extracts.

### RPA1N is important for resection in human cells

**Figure 5.**
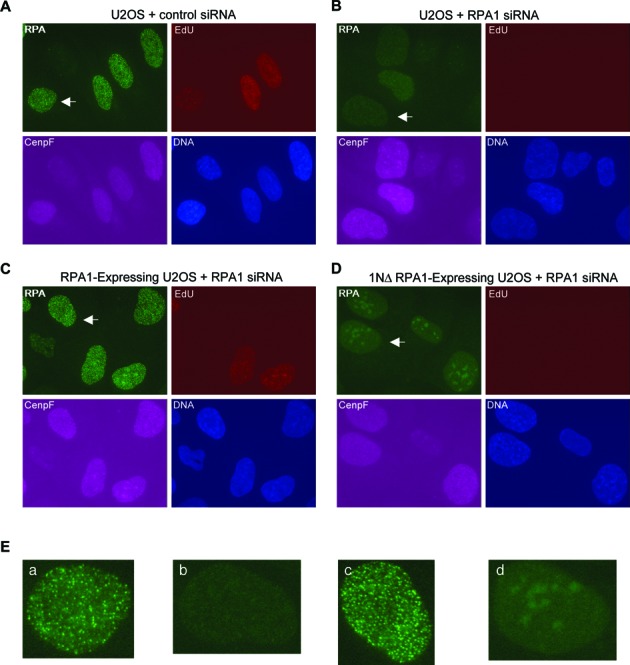
RPA1N is important for resection in cells. (**A**) Etoposide-induced RPA foci in U2OS cells treated with control siRNA. (**B**) Etoposide-induced RPA foci in U2OS cells treated with RPA1 siRNA. (**C**) Etoposide-induced RPA foci in U2OS cells expressing wild-type RPA1 treated with RPA1 siRNA. (**D**) Etoposide-induced RPA foci in U2OS cells expressing RPA1 that lacks the N-terminal domain treated with RPA1 siRNA. The RPA1 siRNA targets the 3′ UTR of the endogenous RPA1 gene, which is not present in the two ectopic RPA1 genes. Cells were treated with different siRNAs for 72 h and then with 250 μM etoposide for 2 h. They were co-stained for RPA, CenpF and EdU. EdU was added to the media 15 min before etoposide. (**E**) Close-ups of the nuclei indicated by arrows in (**A**)–(**D**).

We further determined if the RPA1N domain is also important for resection in cells. To do so, we used siRNAs to specifically knock down the expression of RPA1 in U2OS cells. Resection occurs only in S and G2 cells, so two markers were used to determine the cell cycle stage: EdU, a nucleotide analog incorporated into DNA during S phase, and CenpF, a centromeric protein accumulated during S and G2 phases. Because RPA is essential for DNA replication, DSBs were introduced with etoposide, an inhibitor of Topoisomerase 2 that can induce DSBs independently of DNA replication (26). In control siRNA treated cells, etopoisde induced a large number of discrete RPA foci (detected by an antibody against RPA2) in S and G2 nuclei (Figure 5A and E). In RPA1 siRNA treated cells, most of the cells were EdU^−^ but still CenpF^+^, suggesting that they were, as expected, arrested inside S or G2. As expected, etoposide failed to induce RPA foci in these cells (Figure 5B and E). We then determined if this defect could be complemented by the expression of siRNA-resistant wild-type RPA1 gene or mutant RPA1 gene that lacks the N terminus. Cells carrying these two genes were first treated with the RPA1 siRNA and then with etoposide. As shown in Figure 5C, D and E, while the wild-type RPA1 gene could effectively restore RPA focus induction by etoposide, the mutant RPA1 gene failed to do so. Collectively, these data suggest that the RPA1N domain is also important for resection in cells.

### The spatial location of RPA1N affects RPA's activity to stimulate WRN

**Figure 6.**
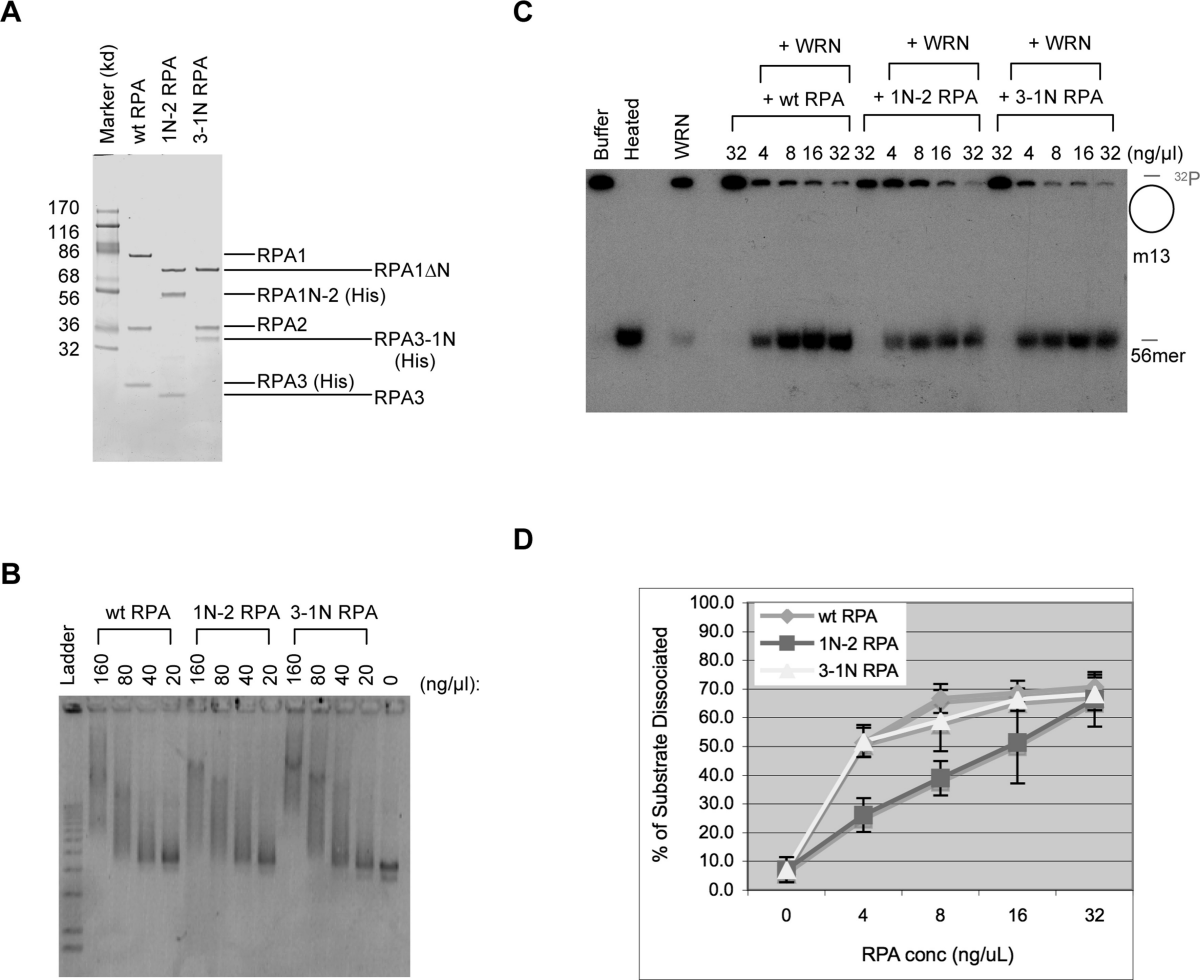
The spatial location of RPA1N affects the stimulation of WRN's helicase activity. (**A**) A gel showing recombinant *Xenopus* wild-type RPA and two mutant RPAs. Proteins were separated by a 4–12% NUPAGE MES gel (Invitrogen, CA, USA) and stained by Coomassie brilliant blue. (**B**) An agarose gel showing the binding activity of the wild-type and mutant RPAs to ss-m13 DNA. (**C**) Effect of wild-type RPA and mutant RPAs on the unwinding activity of WRN. The substrate was a ^32^P-labeled 56mer oligonucleotide annealed to m13 ss-DNA. (**D**) Quantitaton of the unwinding activity in the presence of wild-type RPA or mutant RPAs. The percentages of the oligonucleotide dissociated were quantitated and the averages and standard deviations were calculated from three sets of data and plotted.

RPA's binding to ss-DNA is directional such that the RPA1N domain is positioned at the 5′ end of bound ss-DNA (21,22). Interestingly, during resection WRN moves in the 3′->5′ direction along 3′ ss-DNA to unwind DNA at the ss/ds junction, and DNA2 degrades ss-DNA from the 5′ end. RPA1N is thus optimally positioned to recruit each protein to the respective DNA substrate. The prediction of this hypothesis is that if RPA1N is relocated away from the 5′ end, the RPA complex might no longer be effective in stimulating WRN and DNA2 and in supporting resection. We tested this hypothesis by relocating RPA1N to the N-terminus of RPA2 or the C-terminus of RPA3. The two mutant protein complexes were constructed, expressed in bacteria, and purified (Figure 6A). As expected, both of them still retained normal ss-DNA binding activity (Figure 6B). They were then analyzed for the stimulation of WRN's helicase activity using the same assay as described above (Figure 2). The mutant complex with 3–1N was almost as effective as the wild-type RPA in stimulating WRN's helicase activity (Figure 6C&D). However, the mutant complex containing 1N-2 was less effective, with ca. 60% of the activity of the wild-type RPA even at the highest concentration of protein assayed. These data suggest that the spatial position of RPA1N affects its ability to stimulate WRN's helicase activity.

### The spatial location of RPA1N is important for RPA to stimulate DNA2

**Figure 7.**
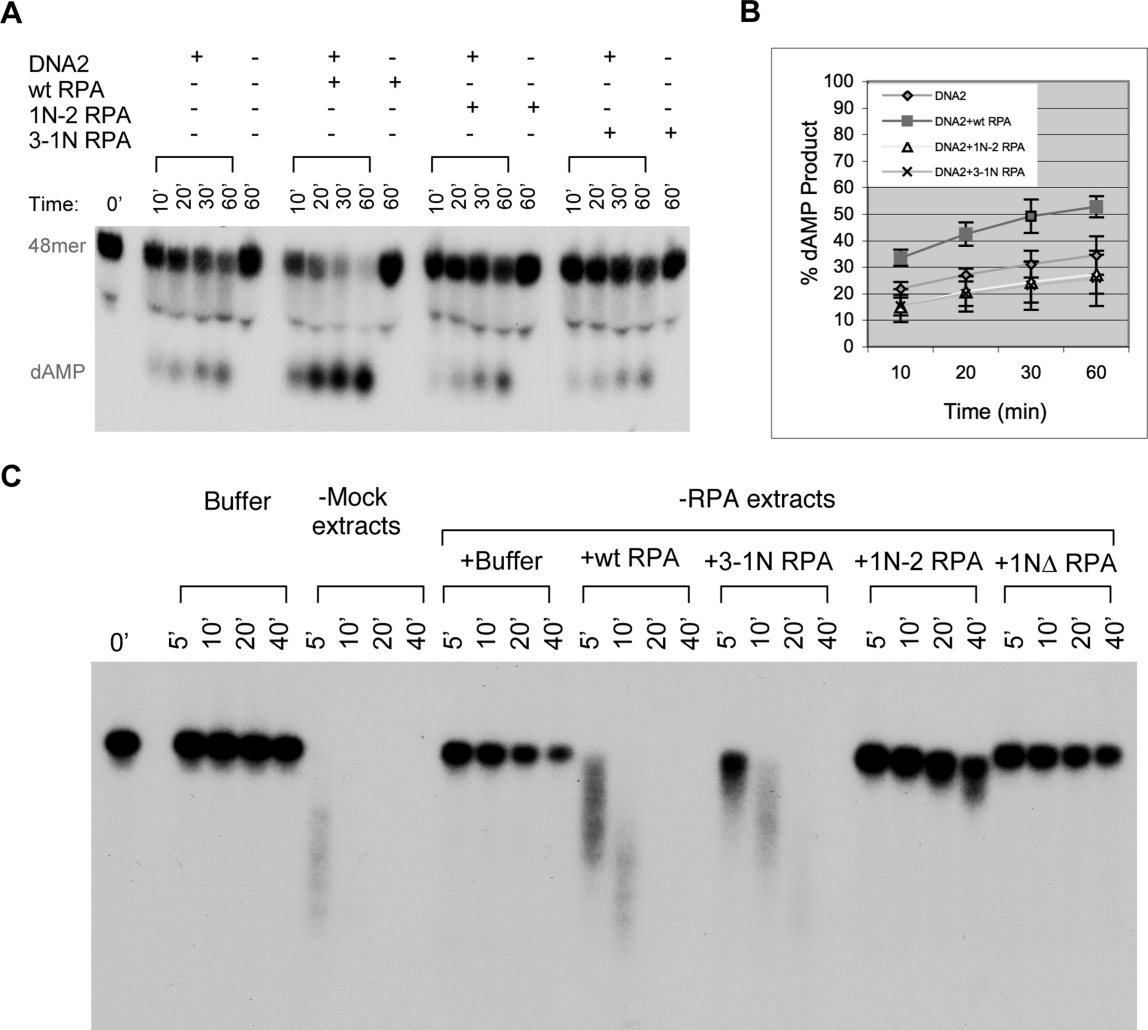
The spatial location of RPA1N is important for stimulating the ss-DNA exonuclease activity of DNA2. (**A**) Nuclease assay with a ^32^P-labeled ss-48mer oligonucleotide attached to magnetic beads via a biotinylated nucleotide at the 3′ end of the labeled strand. The substrate was incubated with DNA2 and wild-type or mutant RPAs (4 ng/μl) for the indicated times and the products were analyzed by a 8% TAE-PAGE. (**B**) The percentages of nucleotide products were quantitated and the averages and standard deviations were calculated from three sets of data and plotted. (**C**) Effect of wild-type and mutant RPAs on the degradation of a 5.7kb ss-DNA with a ^32^P label near the 3′ end in *Xenopus* egg extracts. RPA-depleted extracts were supplemented with buffer or various RPA proteins (20ng/μl). Samples were taken at the indicated times and analyzed by a 1% TAE-agarose gel.

The ability of the mutant RPAs to stimulate DNA2's 5′->3′ ss-DNA exonuclease activity was then assayed using the same method as described for Figure 3. Compared to the wild-type RPA, neither mutant complex could stimulate DNA2's 5′->3′ ss-DNA exonuclease activity (Figure 7A and B). Both mutant RPA complexes were active at inhibiting the 3′->5′ degradation of ss-DNA by DNA2, confirming that their binding to the ss-DNA substrate was normal (Supplementary Figure S1B). However, the substrate for the 5′->3′ nuclease assay was a 48mer oligonucleotide and the amount of DNA2 was limited to a low level so that the stimulatory effect of RPA could be detected. In contrast, *Xenopus* egg extracts could degrade ss-DNA over 5.7 kb in length in a DNA2-dependent reaction (7). Moreover, the concentration of DNA2 in extracts is significantly higher (>100×) than that in the enzymatic assays. Despite the abundance, DNA2 in the extracts is still dependent on RPA, most likely because without it, many other proteins can bind to ss-DNA and prevent DNA2 from accessing the substrate (18). We thus analyzed the activity of the mutant RPA complexes in supporting long-range degradation of ss-DNA in *Xenopus* egg extracts. The substrate was a heat-denatured 5.7kb DNA with ^32^P at the 3′ end. As shown in Figure 7C, the ss-DNA was rapidly degraded in mock depleted extracts. In RPA depleted extracts, it was stable except for that the 3′ ^32^P label was gradually lost due to lack of protection by RPA (18). As expected, the degradation defect could be efficiently rescued by the wild-type RPA. In contrast, the RPA1N deletion mutant, 1NΔ RPA, was inactive in supporting the degradation of ss-DNA, but the 5′ label was now protected, which is consistent with the ss-DNA binding activity of this mutant complex. The 3-1N mutant complex showed activity, but weaker than the wild-type. The 1N-2 mutant complex, on the other hand, was similar to 1NΔ RPA and showed very little activity in supporting ss-DNA degradation. The substrate became slightly faster migrating and the 5′ label was also protected. Collectively, these data suggested that relocating RPA1N to the N-terminus of RPA2 causes a more severe defect than to the C-terminus of RPA3 in stimulating DNA2's 5′->3′ ss-DNA exonuclease activity in *Xenopus* egg extracts.

### The spatial location of RPA1N is important for resection

**Figure 8.**
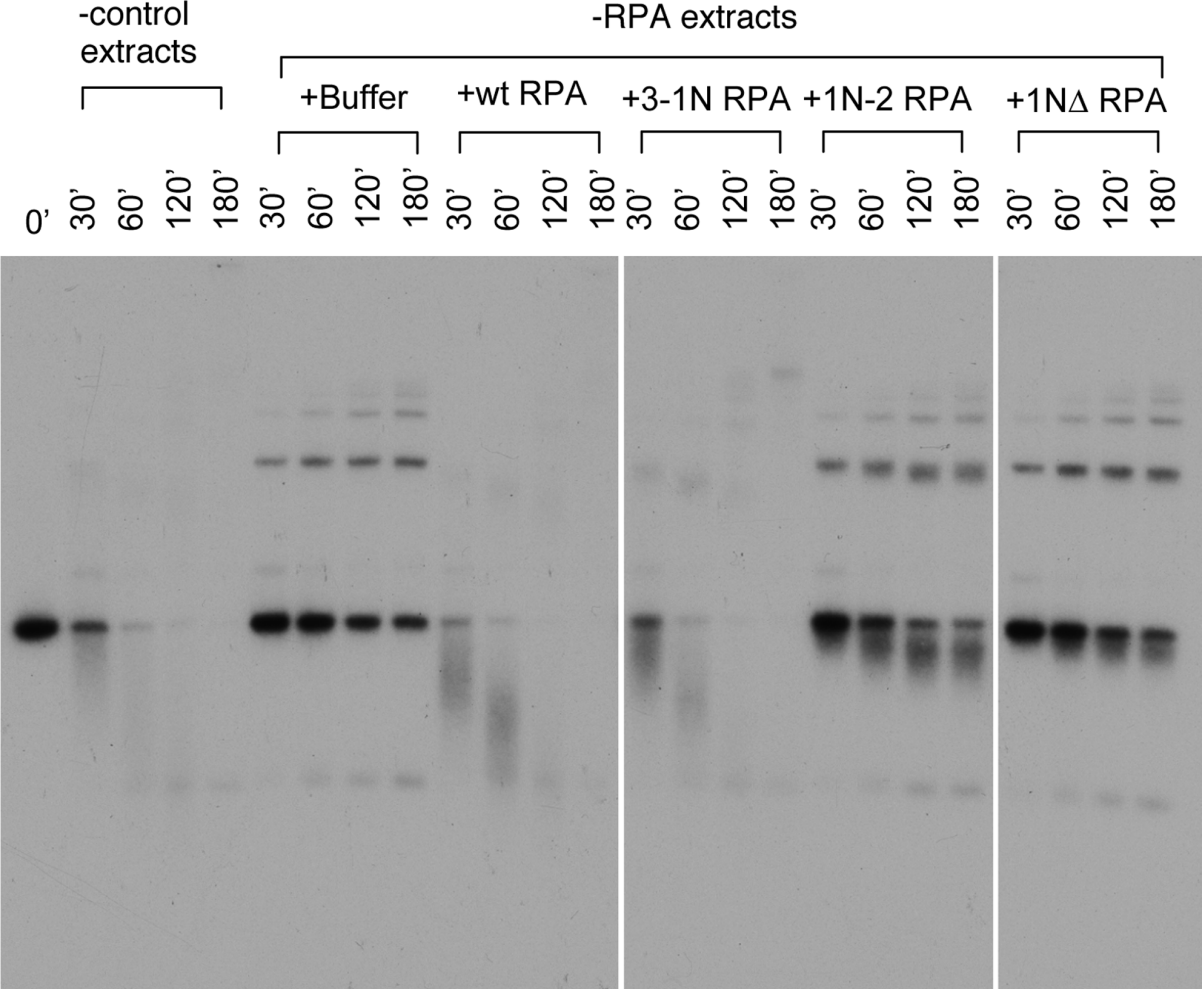
The location of RPA1N is important for the resection of ds-DNA. The substrate for resection was a 5.7 kb linearized ds-DNA with a ddC at the 3′ end and a ^32^P label immediately inside. The substrate was incubated with mock depleted or RPA depleted extracts supplemented with various RPA proteins or buffer. Samples taken at the indicated times were analyzed by a 1% TAE-agarose gel electrophoresis.

What might be the effect of the two mutant RPAs with the relocated RPA1N on resection? To address this question, we used the same assay as used in Figure 4. The 5.7 kb DNA was incubated in depleted extracts supplemented with different RPA complexes or buffer. Samples were taken at various times and analyzed by TAE agarose gel electrophoresis. As shown in Figure 8, the 3-1N mutant complex was active in rescuing resection, which is consistent with its largely intact ability to stimulate WRN and only partially compromised ability to stimulate DNA2. However, the 1N-2 mutant complex was inactive in supporting resection. Even after 3 h of incubation, there was still significant amount of substrate left. Interestingly, there was some faster migrating DNA. This is consistent with the unwinding of a fraction of DNA substrate, presumably due to the significant residual activity of this mutant to stimulate WRN's helicase activity. In support of this, the RPA1N deletion mutant, which had the most severe defect in stimulating WRN and in supporting resection, generated much less faster migrating DNA. Together, these data suggested that the spatial position of the RPA1N domain is indeed important for resection.

## DISCUSSION

A unique feature of DSB resection is the 5′->3′ directionality, which generates 3′ ss-DNA for homology-dependent DSB repair. In this study, we investigated how WRN-DNA2-RPA, the major resection machinery for resection in *Xenopus* egg extracts, accomplishes this directionality. Our major findings are: (i) RPA1N, the N terminus of RPA1, interacts with both WRN and DNA2; (ii) RPA1N is required for the stimulation of WRN's helicase activity; (iii) RPA1N is required for the stimulation of DNA2's 5′->3′ ss-DNA exonuclease activity; (iv) RPA1N is important for resection in *Xenopus* egg extracts; (v) RPA1N is important for resection in human cells; (vi) the spatial location of RPA1N on the RPA complex affects its activity to stimulate WRN and DNA2; and (vii) the spatial position of RPA1N also has a major impact on its ability to support resection. These findings suggest that RPA1N as well as its spatial location are important for positioning WRN and DNA2 to degrade DNA in the 5′->3′ direction.

RPA is a highly conserved protein with a well-defined modular structure (19,20). The N terminal 120 amino acids of human RPA1 form a distinct domain that has no significant affinity for DNA but can interact with many other proteins such as p53 and checkpoint proteins (19,20,24,29). Our finding that the N terminal 121 amino acids of *Xenopus* RPA1 (corresponding to amino acids 1–121 of human RPA1) can interact with *Xenopus* WRN and DNA2 suggests that they form the same structurally independent domain for protein–protein interaction. A smaller region encompassing amino acids 1–113 cannot interact with WRN, but the GST fusion protein had low solubility (unpublished data). This was again in agreement with the human RPA studies that 120 amino acids are required for structural integrity of RPA1N (29). As such, the inability of amino acids 1–113 to interact with WRN is most likely due protein mis-folding. Interestingly, this domain also interacts with many other proteins involved in replication, repair, and checkpoint activation (19,20). Future studies are required to determine if WRN, DNA2, and others interact with RPA1N via one or multiple surface patches.

The functional importance of RPA1N is supported by the enzymatic data and the resection assays. A mutant RPA complex that lacks RPA1N possesses normal ss-DNA binding activity, but loses the ability to stimulate the WRN's helicase activity and DNA2's nuclease activity. It also loses the ability to support the resection of long DNA molecules in *Xenopus* egg extracts. In U2OS cells treated with siRNAs that silence the expression of RPA1, the expression of a mutant RPA1 lacking the RPA1N is also unable to support resection of DSBs induced by etoposide. This result is different from a previous study reporting that RPA1N is not required for the localization of RPA to discrete foci induced by various DNA damaging drugs including etoposide (30). Likely explanations for this discrepancy are the cell lines and siRNAs used. We used U2OS cells instead of HeLa cells and the siRNA knockdown appeared to be much faster (after 48 h versus 96 h). Further studies are required to resolve this issue.

**Figure 9.**
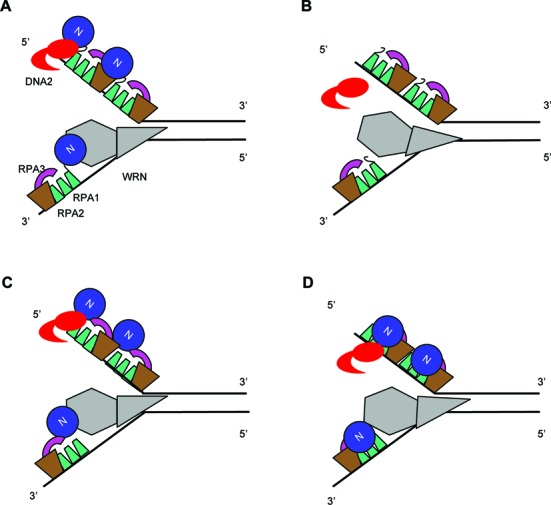
Model of the position of RPA1N and stimulation of WRN's 3′->5′ helicase activity and DNA2's 5′->3′ exonuclease activity. (**A**) RPA1N in the wildtype complex is optimally positioned to interact with WRN at the ss/ds-DNA junction on the 3′ strand and with DNA2 approaching from the 5′ end. RPA binding to the rest of ss-DNA prevents the reannealing of the unwound strands and the endonuclytic cleavage by DNA2. (**B**) 1NΔ RPA can still bind to ss-DNA to protect it against endonuclytic cleavage by DNA2 and to facilitate unwinding by preventing reannealing of the single strands unwound by the low intrinsic helicase activity of WRN. (**C**) RPA1N in the 3–1N RPA complex is still positioned close enough to interact normally with WRN and DNA2 and stimulate their activities. (**D**) RPA1N in the 1N-2 RPA complex is shifted more drastically in space and is less effective to recruit WRN to the ss/ds-DNA junction. It is completely incapable of recruiting DNA2 to the 5′ end of ss-DNA.

The most unique finding of this study is that not only RPA1N but also its spatial location are important for function. RPA binds to ss-DNA in a directional way so that the N terminus of RPA1 is situated at the 5′ end, while DNA bindings domains A-C in RPA1 and D in RPA2 lay down sequentially on ss-DNA towards the 3′ end. This orientation is ideal for making contact with WRN, which is positioned at the ss-DNA/ds-DNA junction and moves along the 3′ strand to unwind DNA. It is also optimal for interacting with DNA2 to promote its attack on ss-DNA from the 5′ end (Figure 9). Similar orientations have been proposed to facilitate the recruitment of various nucleotide excision repair proteins and checkpoint proteins to the respective target DNA structures, but this hypothesis has never been experimentally tested (21,23,24). Our study represents the first demonstration that the spatial location of RPA1N is indeed important for the function of RPA. RPA1N is connected to the rest of the complex by a flexible linker and is thus omitted from structural studies (31,32). Based on the structure of Ustilago RPA bound to ss-DNA, moving RPA1N to the C-terminus of RPA3 would shift it laterally by ca. 20 Å to the middle region between the 5′ and 3′ ends of the bound ss-DNA (31). It is still in a similar peripheral region of the RPA complex. The flexibility of the linker connecting RPA1N to DBD-A suggests that the actual shift in space might be even less. Consistent with the structural inference, the mutant RPA complex with 3–1N is quite active in stimulating WRN's helicase activity and only partially defective in stimulating DNA2's nuclease activity. In egg extracts, which contain significantly more DNA2 protein than in the enzymatic assays, this mutant is still capable of supporting resection. On the other hand, moving RPA1N to the N-terminus of RPA2 complex causes a more radical shift in space. It is now juxtaposed to the trimeric core and much closer to the 3′ end instead of the 5′ end of the bound ss-DNA. Consistent with this structural inference, the 1N-2 mutant RPA complex is partially defective in stimulating WRN's helicase activity, completely inactive in stimulating the DNA2's 5′->3′ ss-DNA exonuclease activity, and unable to support resection. Overall, these data strongly suggest that the spatial location of RPA1N is a critical factor for determining the 5′->3′ directionality of the WRN-DNA2-RPA resection pathway.

## Supplementary Material

SUPPLEMENTARY DATA
