# Supplementary material for: The N-terminus of RPA large subunit and its spatial position are important for the 5′->3′ resection of DNA double-strand breaks
Source: Nucleic Acids Res. 2015 Oct 10;43(18):8790–800. doi: 10.1093/nar/gkv764 (PMC4605295; doi:10.1093/nar/gkv764)
Supplement: SUPPLEMENTARY DATA [file supp_43_18_8790__index.html]

The N-terminus of RPA large subunit and its spatial position are important for the 5′->3′ resection of DNA double-strand breaks — The N-terminus of RPA large subunit and its spatial position are important for the 5′->3′ resection of DNA double-strand breaks — SUPPLEMENTARY DATA 

# The N-terminus of RPA large subunit and its spatial position are important for the 5′->3′ resection of DNA double-strand breaks

## SUPPLEMENTARY DATA

- SUPPLEMENTARY DATA
