# Supplementary material for: The N-terminus of RPA large subunit and its spatial position are important for the 5′->3′ resection of DNA double-strand breaks
Source: Nucleic Acids Res. 2015 Oct 10;43(18):8790–800. doi: 10.1093/nar/gkv764 (PMC4605295; doi:10.1093/nar/gkv764)
Supplement: SUPPLEMENTARY DATA [file supp_gkv764_nar-01496-d-2015-File011.pdf]

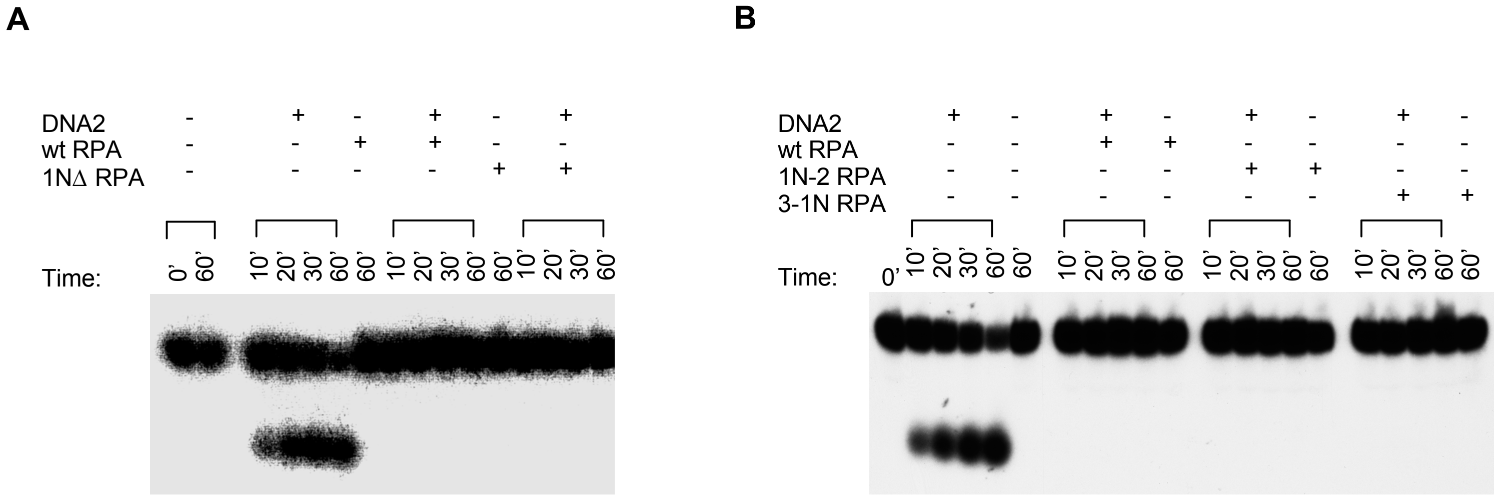

Figure S1. Mutant RPA complexes retain the activity to protect the 3' end of ss-DNA from DNA2 attack. (A) Effect of wild-type and 1NΔ RPA on the 3'->5' degradation of ss-DNA by DNA2. The substrate was a 3' ^{32}P-labeled ss-48mer oligonucleotide attached to magnetic beads via a biotinylated nucleotide at the 5' end. It was incubated with DNA2 and wild-type or 1NΔ RPA (4/ng/µl) for the indicated times and the products were analyzed by a 8% TAE-PAGE. The gel was dried and exposed to Fuji phosphorimager. (**B**) Effect of 1N-2 and 3-1N RPA on the 3'->5' degradation of ss-DNA by DNA2. The substrate was incubated with DNA2 and wild-type, 1N-2, or 3-1N RPA (4/ng/µl) for the indicated times and the products were analyzed by a 8% TAE-PAGE. The gel was dried and exposed to X-ray film.

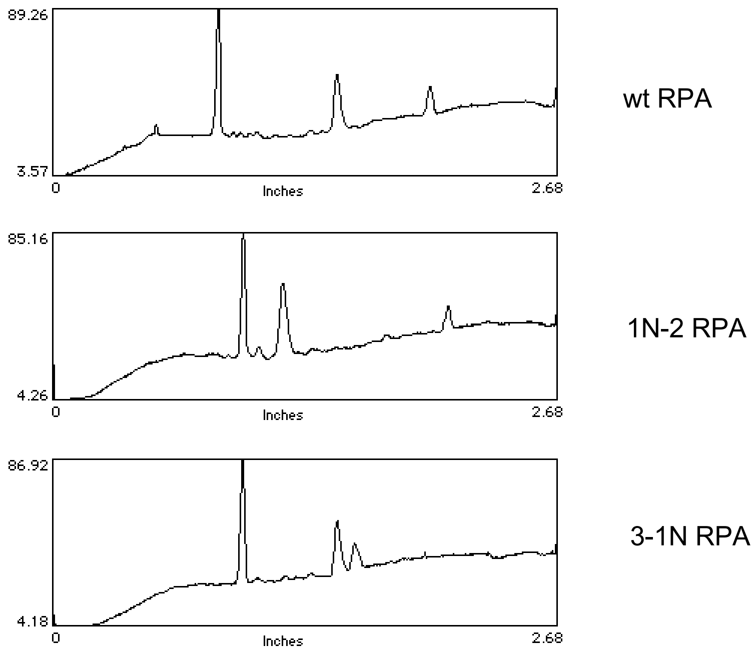

Figure S2. Density plots of the wild-type, 1N-2, and 3-1N RPA complexes. The gel in **Figure 6A** was scanned and plotted using NIH image. Comparisons were made between the wild-type and 3-1N, which share the RPA2 subunit, and between 3-1N and 1N-2, which share the RPA1ΔN subunit.
